# Genomic instability in mutant p53 cancer cells upon entotic engulfment

**DOI:** 10.1038/s41467-018-05368-1

**Published:** 2018-08-03

**Authors:** Hannah L. Mackay, David Moore, Callum Hall, Nicolai J. Birkbak, Mariam Jamal-Hanjani, Saadia A. Karim, Vinaya M. Phatak, Lucia Piñon, Jennifer P. Morton, Charles Swanton, John Le Quesne, Patricia A. J. Muller

**Affiliations:** 1 MRC Toxicology Unit, Lancaster Road, Leicester, LE1 9HN UK; 2 Cancer studies, University of Leicester, Leicester, LE1 7RH UK; 3 Cancer Research UK Manchester Institute, The University of Manchester | Alderley Park, Manchester, SK10 4TG UK; 4 Translational Cancer Therapeutics Laboratory, The Francis Crick Institute, 1 Midland Rd, London, NW1 1AT UK; 5 Cancer Research UK Lung Cancer Centre of Excellence, University College London Cancer Institute, Paul O’Gorman Building 72 Huntley Street, London, WC1E 6BT UK; 6 Department of Medical Oncology, University College London Hospitals, 235 Euston Rd, Fitzrovia London, NW1 2BU UK; 7 CRUK The Beatson Institute, Garscube Estate, Switchback Road, Glasgow, G61 1BD UK; 8 Institute of Cancer Sciences, University of Glasgow, Switchback Road, Glasgow, G61 1BD UK; 9 Department of Histopathology, Glenfield Hospital, University Hospitals Leicester NHS Trust, Groby Road, Leicester, LE3 9QP UK; 10 Present Address: Institute of Cancer and Genomic Sciences, University of Birmingham, Birmingham, B15 2TT UK

## Abstract

Cell-in-cell (CIC) structures are commonly seen in tumours. Their biological significance remains unclear, although they have been associated with more aggressive tumours. Here we report that mutant p53 promotes CIC via live cell engulfment. Engulfed cells physically interfere in cell divisions of host cells and for cells without p53 this leads to host cell death. In contrast, mutant p53 host cells survive, display aberrant divisions, multinucleation and tripolar mitoses. In xenograft studies, CIC-rich p53 mutant/null co-cultures show enhanced tumour growth. Furthermore, our results show that CIC is common within lung adenocarcinomas, is an independent predictor of poor outcome and disease recurrence, is associated with mutant p53 expression and correlated to measures of heterogeneity and genomic instability. These findings suggest that pro-tumorigenic entotic engulfment activity is associated with mutant p53 expression, and the two combined are a key factor in genomic instability.

## Abstract (web summary)

Cell-in-cell (CIC) structures are frequently observed in cancers; however, it is unclear how their formation contributes to tumorigenesis. Here, the authors show that the high frequency of CIC is linked to mutant p53 status and CIC containing p53 mutant cancer cells are pro-tumorigenic due to enhanced genomic instability.

## Introduction

There has been a recent growth in research focusing on cell-in-cell (CIC) structures in tumours, which is starting to provide new insights into their mechanism of formation and biological implications. CIC structures represent one viable cell existing within the membrane of another^1^, and have been recognised in human tumour tissues for over a century^2^. In a key study, Overholtzer et al.^3^ described a process of in-cell invasion, entosis, as a route to non-apoptotic cell death via CIC formation. At other times, different names have been given to processes causing CIC including cannibalism^4^, emperipolesis^5^, and cell engulfment^6^ that subtly differ in which cell is driving the event, under what circumstances the event is happening and what types of cells are being internalised. In the context of cancer biology, CIC formation is a suggested mediator of cell competition, which could ultimately have either pro-tumorigenic or anti-tumorigenic consequences^7^.

CIC structures have been identified in a number of solid tumours, including breast, lung, endometrial, pancreatic, skin, and oral cancers^8–13^. In effusion and urine cytology, CIC structures are specific diagnostic indicators of malignant processes^14^. Links between histological grade and CIC have also been described in breast^8^ and urothelial carcinoma^15^. Collectively, these suggest a pro-tumorigenic association with CIC structures, but no causal link between CIC and tumour growth or other phenotypes have yet been shown.

We have chosen lung adenocarcinoma as our model in which to investigate the potential impact of CIC formation on tumorigenesis. Lung cancer remains the leading cause of cancer related death worldwide^16^ with adenocarcinoma, the most common type, accounting for 40% of cases^17^. CIC structures have been observed in both small cell lung cancer-derived cell lines and primary giant cell tumours^9,18^ but have not been described in lung adenocarcinoma, in which their prevalence and clinico-pathological significance is unknown.

Large cohort studies of lung adenocarcinoma have mapped a number of common driving genomic events^19^. TP53 mutations are found in around half of non-small cell lung cancers^20^ and are very common in many other tumour types^21^. The p53 protein is a tumour suppressor involved in regulating the expression of hundreds of genes that control a variety of cellular processes including apoptosis, cell cycle check points and cell senescence^22^. When mutated, p53 expression is either lost or a mutant protein is expressed that has often lost the tumour suppressive functions of wild-type (WT) p53. More remarkably, these mutant proteins generally acquire novel functions in promoting tumour growth, invasion, and chemoresistance. These functions are termed ‘gain-of-function’ and are independent of any remaining WT p53^23^.

In this study we discovered that mutant p53 expression could promote the formation of CIC structures in cell lines and that mutant p53 status is associated with increased CIC occurrence in lung adenocarcinoma. We further explored the consequences of CIC both for the individual cell and for tumours formed as xenografts in recipient mice. Our results suggest that entotic engulfment is associated with mutant p53 expression, promotes tumorigenesis and disease recurrence, and facilitates abnormal mitotic events, which are linked to genomic instability.

## Results

### CIC formation is driven by mutant p53 expression

**Fig. 1:**
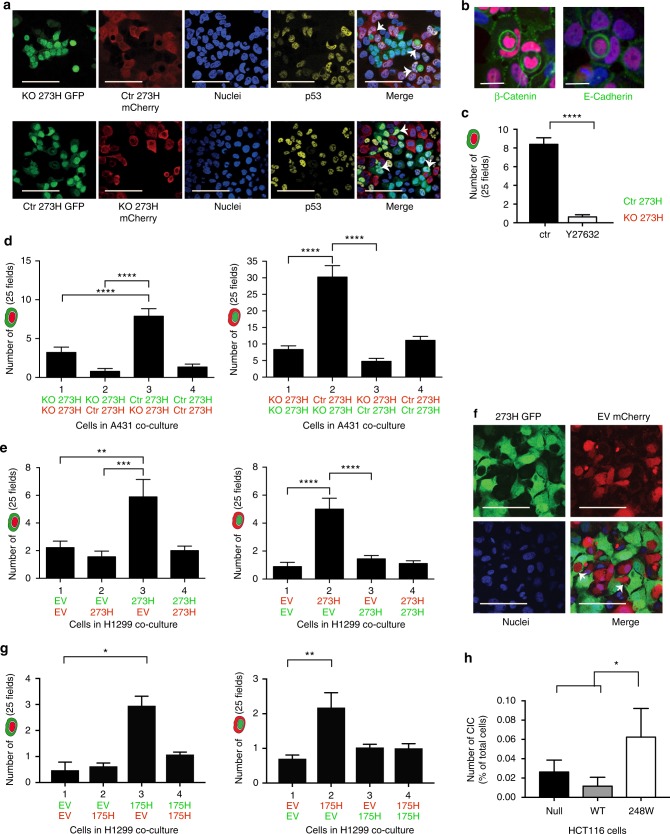
CIC occurrences are predominantly seen in mutant p53 cells. **a** Confocal images of CIC formed in A431 with a CRISPR control plasmid (Ctr 273H) or CRISPR p53 knock out (KO 273H) cells (in addition to fluorescent constructs as indicated). Arrows indicate CIC, p53 (yellow), nuclei (Dapi). Scale bars = 100 μm. **b** Immune fluorescent staining of β-Catenin (left) or E-Cadherin (right) in green, p53 in red and Dapi in blue. Scale bars = 50 μm. **c** Number of CIC events in mutant p53 (green)/KO p53 (red) co-cultures in which engulfment was quantified with or without Y27632 (5 μM) after 48 h. Each bar represents +/−SEM of triplicate experiments ****p < 0.0001. **d** Quantification of CIC in A431 cells. Co-cultures were seeded in the following combinations; 1: KO 273H/mCherry with KO 273H/eGFP, 2: KO 273H/eGFP with Ctr 273H/mCherry, 3: Ctr 273H/eGFP with KO 273H/mCherry and 4: Ctr 273H/eGFP with Ctr 273H/mCherry. Total numbers of CIC occurrences are shown where the outer engulfing cell is green (left graph) or red (right graph). Each bar represents +/−SEM of triplicate experiments ****p < 0.0001. **e** Quantification of CIC in four different co-cultures of H1299 cells transfected with EV or 273H along with eGFP or mCherry; 1 EV/eGFP with EV/mCherry, 2 EV/eGFP with 273H/mCherry, 3 EV/mCherry with 273H/eGFP and 4 273H/eGFP with 273H/mCherry. Graphs show CIC with occurrences for each condition based on whether the outer engulfing cell was green or red as indicated by cartoons in the legend. Error bars represent SEM of triplicate experiments **p = 0.0011, ***p = 0.0002 ****p < 0.0001. **f** Confocal images of CIC (indicated with arrows) in green H1299 cells (transfected with R273H mutant p53 and eGFP) and red H1299 cells (transfected with EV and mCherry). Nuclei (DAPI) in blue. Scale bars = 50 μm. **g** Same as **e**, but with the 273H mutant p53 H1299 cell lines replaced with 175H mutant p53. Each bar represents +/−SEM of triplicate experiments *p = 0.048, **p = 0.008 **h** Percentage engulfment in HCT116 null, WT or mutant p53 R248W cells. Each bar represents +/−SEM of triplicate experiments *p = 0.016 (null vs R248W) or *p* = 0.012 (WT vs R248W)

While generating fluorescent cells to study the differences between mutant p53 and p53 null cells, we noted that these cells often interacted with each other and that one cell type often engulfed the other leading to so called CIC structures. To investigate this in more detail, we used A431 (p53 273H) cells that were transfected with either eGFP or mCherry plasmids or CRISPR constructs to knock out p53. This allowed cells with differing p53 status to be mixed and co-cultures to be followed in time-lapse microscopy. CIC structures were visible after 2–5 days of co-culturing and appeared to be formed via an engulfment process with one cell engulfing around another (Fig. 1a, Supplementary Fig 1a and Supplementary Movie 1).

Upon close examination, engulfment of our cells displayed many of the features of the recently described process of entotic engulfment that relies on cell contractility of the internalised cell by RhoA and ROCK signaling and cell–cell adhesion through β-catenin and E-Cadherin^3^. Treatment of A431 cells with the ROCK inhibitor Y27632A almost completely abolished engulfment (Fig. 1c). Furthermore, an increased expression of E-Cadherin and β-catenin on the membrane between the inside cell and the outer cell was detected (Fig. 1b) that was not seen on the outside membrane shared with neighbouring cells (Fig. 1b and Supplementary Fig. 1b, c). Cells that were being engulfed appeared to be rounded up due to just entering or leaving mitosis (Supplementary Fig. 1a), which was also observed to happen in entotic cell engulfment^24^. Previous reports suggest a role for glucose starvation in entosis, but lowering glucose levels did not promote engulfment by mutant p53 cells (Supplementary Fig. 1d), but rather decreased it. Mutant p53 cells promote glucose import by regulating the Glut1 receptor^25^ and glucose deprivation has been shown to decrease mutant p53 expression^26^. These results suggest that mutant p53 cells rely heavily on glucose for survival suggesting that glucose starvation cannot trigger entotic engulfment in mutant p53 cells.

Interestingly, A431 cells R273H mutant p53 cells were more often observed to be the outer ‘host’ cell in the final CIC structure than mutant p53 KO cells (Fig. 1a). This was quantified by observing how often an outer cell was green (Fig. 1d, left) or red (Fig. 1d, right) in different p53 co-culture conditions. p53 null cells were able to engulf, but when combined with mutant p53 in heterogeneous populations (co-cultures 2 and 3) mutant p53 cells were significantly more often seen to be the external ‘host’ cell (Fig. 1d). There was a slight preference for mCherry transfected cells to engulf eGFP transfected cells, which most likely is due to variations in colour intensities (compare CIC numbers in KO/KO conditions between left and right figure in Fig. 1d). When total entotic engulfment events in each condition were combined, we detected a two-fold increase in the total number of CIC in the heterogeneous populations 2 and 3 compared to the p53 KO homogeneous population (Fig. 1d). More remarkably, the mutant p53 homogenous population did not differ much in total engulfments compared to the KO homogenous population (Supplementary Fig. 1e). Taken together, mutant p53 cells display entotic cell engulfment that, similar to entosis, relies on competition between cells^27^ with an advantage for mutant p53 cells to engulf ‘weaker’ p53-null neighbours, but not competitor mutant p53 cells

To verify our A431 results (Fig. 1d) in another cell type, we created fluorescent mutant p53 expressing H1299 cells (273H) to co-culture with cells transfected with an empty vector (EV) in combinations indicated in Fig. 1e, f. As with A431 cells, mutant p53 status promoted engulfment with heterogeneous populations showing higher rates of entotic engulfment (Fig. 1d–f, Supplementary Fig. 2c and Supplementary Movie 2). p53 mutations occur throughout the p53 protein and have been found at nearly every amino acid^28^. To study if other p53 mutations also promoted CIC formation, we generated fluorescent mutant p53 R175H H1299 cells and similarly detected an enhanced engulfment in heterotypic cultures that was not seen in the homotypic cultures (Fig. 1g and Supplementary Fig. 1h). Transient transfection of R175H, R248W or R273H (in combination with mCherry) in A431 p53 KO cells also promoted engulfment, compared to cells transfected with mCherry alone, confirming that different mutant p53 proteins promote entotic engulfment in a similar background (Supplementary Fig. 1i.)

In order to assess whether WT p53 was able to promote entotic engulfment, we attempted to express WT p53 in A431 KO cells. However, in the time frame required to study entosis, WT p53 initiated cell death in a large proportion of cells. We therefore resorted to using a transcriptionally inactive p53 construct (22/23) in A431 p53 KO cells, to prevent p53 mediated cell death or cell senescence. Expression of this variant did not promote entotic engulfment, but expression of mutant p53 R273H, or other mutants such as R175H or R248W did significantly promote entotic engulfment (Supplementary Fig 1i). In addition, HCT116 cells (WT p53) or HCT116 p53 null cells showed much less CIC structures than HCT116 mutant p53 (mutant 248W) cells (Fig. 1h), indicating that WT p53 is not promoting entotic engulfment under normal conditions.

Engulfment was also evident in other cell lines; BxPC3 (Y220C mutant p53), H358 (p53 null), and H322m (R248L mutant p53) cell lines (Supplementary Fig 1j), but not in U2OS (WT p53), PC-9 (WT p53) or A549 (WT p53) (Supplementary Fig. 1j, k). Interestingly, different cell lines displayed varying degrees of entotic engulfment capacity dependent on the number of cells that were plated. A431 cells clearly appeared to engulf best when more confluent, while BXPC3 cells lost the ability to engulf when less confluent, making it difficult to compare rates of engulfment between cell lines directly. However, our results are indicative that entotic engulfment is not restricted to one particular cell type and is more common in mutant p53 cell lines.

### Entosis is associated with EGFR and integrin expression

**Fig. 2:**
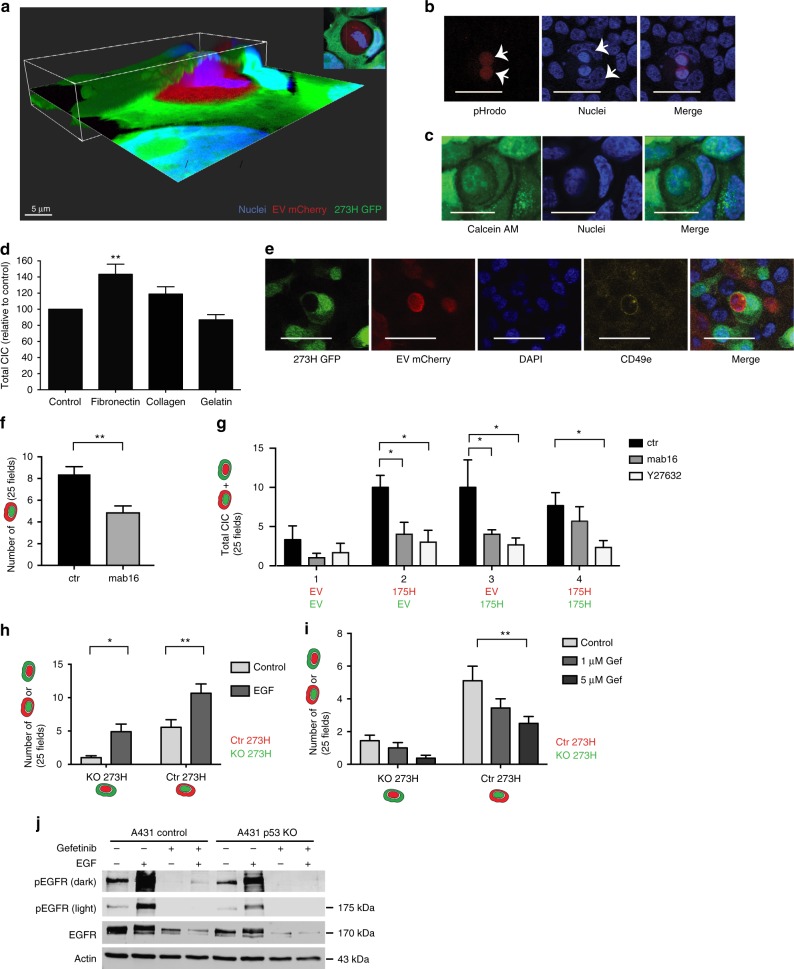
Cell surface molecules regulated by mutant p53 are involved in CIC formation. **a** 3D images of a CIC structure in H1299 cells. The top right insert is showing the horizontal plane by confocal imaging. Scale bar = 5 μm. **b** Fluorescent image of live A431 cells that have been labeled with pHrodo. Scale bars = 50 μm. **c** Confocal image of A431 cells that have been stained with Calcein AM. Scale bars = 20 μm. **d** Quantification of CIC for H1299 co-cultures seeded on plastics coated with different matrices. Each bar represents +/−SEM of triplicate experiments **p = 0.0072. **e** Immunofluorescence image of a CIC structure formed between H1299 cells (green and red as indicated). Dapi is in blue and CD49e (integrin α5) is in yellow. Scale bar = 50 μm. **f** Number of CIC events in mutant p53 (green)/KO p53 (red) A431 co-cultures in which engulfment by red cells was quantified in the presence of mAb16. Each bar represents +/−SEM of triplicate experiments **p = 0.003. **g** Quantification of CIC in H1299 EV/H1299 175H co-cultures in the presence or absence of Y27632 or mAb16. Each bar represents +/−SEM of triplicate experiments *p = 0.01–0.04. **h** Quantification of total CIC structures per 20 hpf observed in A431 Ctr 273H/mCherry and KO 273H/GFP co-cultures treated with and without EGF for 24 h. Either red or green engulfing cells were quantified for KO 273H or ctr 273H respectively. Each bar represents +/−SEM of triplicate experiments Each bar represents +/−SEM of triplicate experiments *p = 0.03 or **p = 0.004. **i** Quantification of total CIC structures per 20 hpf observed in A431 Ctr 273H/mCherry and KO 273H/GFP co-cultured cells treated with and without Gefitinib (24 h). Either red or green engulfing cells were quantified for KO 273H or ctr 273H respectively Each bar represents +/−SEM of triplicate experiments **p = 0.0019. **j** Western blot showing pEGFR and EGFR following treatment of A431 control and p53 CRISPR KO cells, with and without Gefitinib and EGF. Actin was used as loading control

**Fig. 3:**
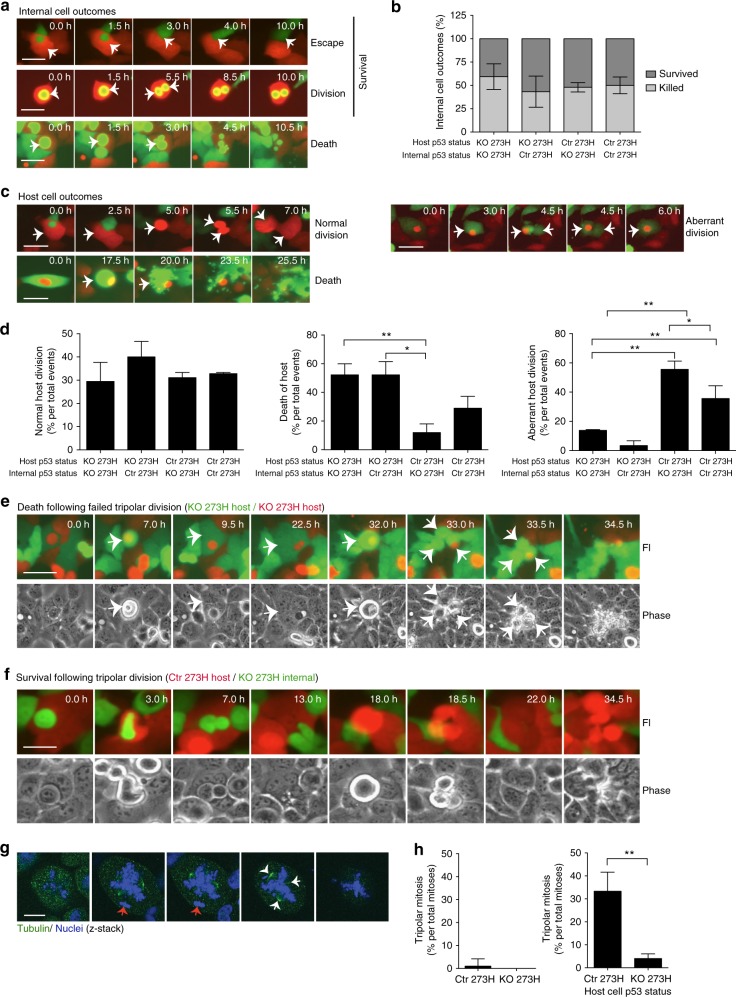
Mutant p53 status determines CIC outcomes. **a** Fluorescent time-lapse images illustrating the internal A431 cell outcomes. Arrows indicate the internal cell that is either escaping (top), dividing (middle) or dying (bottom). Scale bars = 50 μm. (also see Supplementary Movies 3–5) **b** Quantification of CIC outcomes for the internal cell, death or survival, based on the host cell p53 status. Each bar represents +/−SEM of three sets of 20 videos. **c** Fluorescent time-lapse images illustrating host cell outcomes in which arrows indicate activity of the host cell including normal division (top, please note that this is part of the same video as for 3ai), death (bottom) or aberrant division (right). Scale bars = 50 μm. Events indicated with arrows. (also see Supplementary Movies 3, 6 and 7) **d** Quantification of the host cell outcomes following engulfment. This includes normal cell division (left), death (middle) *p = 0.0111, **p = 0.0065 and aberrant division (right) *p = 0.0409, **p = 0.0295. Each bar represents +/−SEM of three sets of twenty videos. **e**, **f** Fluorescent and phase contrast time-lapse images of tripolar division events of a **e** CRISPR KO 273H host and **f** Ctr 253H mutant p53 host, both of which engulfed a CRISPR KO 273H internal cell. White arrows indicate daughter cells, blue arrow indicates where a daughter cell survives and subsequently divides. Scale bars = 50 μm. (please see Supplementary Fig. 2d on this figure with superimposed nuclei). These time lapses correspond to Supplementary Movies 8 and 9. **g** Non-fluorescent A431 mutant p53 cells were stained for tubulin and imaged in a Z-stack with confocal microscopy. Red arrows indicate an engulfed cell, white arrows indicate spindle formation. Scale bar = 20 μm. **h** Quantification of (left) tripolar mitoses as a percentage of total mitoses in Ctr 273H A431 or CRISPR KO A431 cells (error bar represents +/−SEM in ten sets of ten videos) and (right) tripolar mitoses of engulfing cells (error bar represents +/−SEM of seven sets of ten videos) as a percentage of total engulfing cells undergoing mitosis (*n* = 100). Error bars indicate SEM **p = 0.0089

To prove that entotic engulfment was complete and not the result of cells growing on top of each other, Z-stack images were generated of CIC structures that confirmed complete enclosure of the engulfed cell (Fig. 2a). A pH sensitive pHrodo dye^29^ was shown to encompass the engulfed cell, further confirming full engulfment and, in agreement with literature, suggest internalization of the cell in a lysosomal compartment^3^ (Fig. 2b). In most cases the internal cells were alive as Calcein AM staining was visible in many engulfed cells^30^ (Fig. 2c) and internalised cells were able to divide (Fig. 3aii and 3ci).

Our previous work demonstrated a role for mutant p53 in regulating integrin and epidermal growth factor receptor (EGFR) signaling to promote invasion and metastasis^31^. Integrins and EGFR have been shown to promote the related process of phagocytosis that occurs in various immune cells to clear damaged or infected cells^32–34^. Furthermore, ROCK signaling, which others and we have shown to be inhibiting entotic engulfment (Fig. 1c), is a downstream target of integrins^35^. When grown on fibronectin, H1299 cells, but not A431 cells, revealed a small increase in CIC structure formation (Fig. 2d) and integrin (alpha 5 chain) was found to be high expressed on the cell membranes that were shared between the engulfing and engulfed cell (Fig. 2e). The involvement of alpha 5 integrins was further demonstrated by inhibition with a monoclonal antibody (mab16) against the alpha 5 subunit of integrin in A431 cells or with mab16 or Y27632 in H1299 (175H and EV) cells (Figs. 1c, 2f, g). In both cell lines a clear decrease in engulfment was seen upon integrin or ROCK inhibition. Interestingly, the co-cultures of H1299 cells furthermore demonstrated that even homotypic cultures of EV or mutant cells could be inhibited, suggesting that mutant p53 activates engulfment through integrin activation. Similar to integrin inhibition, EGFR inhibition, with gefitinib, resulted in a decrease in entotic engulfment of both mutant p53 cells and p53 null A431 cells (Fig. 2i). Conversely EGF treatment promoted the number of CIC structures in both co-cultures more than 3-fold (Fig. 2h), indicating that entotic engulfment can be induced by EGF in a mutant p53-independent manner. Increased CIC formation coincided with an increased phosphorylation of EGFR that, in agreement with our previous work^31^, was more pronounced in mutant p53 cells than in p53 null cells (Fig. 2j). Together these data suggest a role for mutant p53 mediated activation of integrins and EGFR in cell engulfment.

### CIC formation disturbs cell division of the host cell

In order to understand what the consequences of mutant p53 driven entotic engulfment are for a cell, we monitored co-cultures of our control R273H mutant p53 A431 and CRISPR p53 KO A431 cells. A431, as opposed to H1299 cells, were used as they engulf more frequently and at much lower confluency, which is more suitable to view in 2D time-lapse imaging. Three different outcomes for the engulfed internal cell were seen: escape (Fig. 3a left and Supplementary Movie 3), cell division (Fig. 3a middle and Supplementary Movie 4) or cell death (Fig. 3a right, Supplementary Movie 5). As escape often followed division of the internal cell, these outcomes were quantified together as surviving cells (Fig. 3b). Both mutant p53 and p53 null cells survived about half of all events, regardless of the status of the outside cell (Fig. 3b), suggesting that the outside cell status does not dictate the fate of the inside cell. Three different outcomes for the host cell were noted: normal division (Fig. 3c left top, Supplementary Movie 3), death (Fig. 3c left bottom, Supplementary Movie 6) and aberrant divisions (Fig. 3c right, Supplementary Movie 7). The number of normal divisions was similar in all conditions (Fig. 3d left). Most strikingly, cell death was almost three times higher in p53 null host cells than mutant p53 host cells that were both engulfing p53 KO cells (Fig. 3d middle). Notably, this cell death did not always lead to death of the internal cell, which sometimes escaped. Conversely to cell death, aberrant divisions were seen three times more often in mutant p53 host cells than p53 KO host cells engulfing p53 KO cells (Fig. 3d right). Mutant p53 cells engulfing neighbouring mutant p53 cells were more likely to undergo failed division, but less frequently than a mutant p53 cell engulfing a p53 KO cell (Fig. 3d right). Similarly, the number of dying host cells was less than p53 KO cells, but more than mutant p53 cells engulfing a p53 KO cell. In conclusion, these data suggest that the increased CIC that was seen in heterogeneous mutant p53/p53 null cell populations is at least to an extent caused by increased death of p53 null cells upon engulfment and survival of p53 mutant cells.

Remarkably, cell death of p53 null cells almost always followed a failed division of a p53 KO host (Fig. 3e and Supplementary Fig. 2a, Supplementary Movie 8), whereas mutant p53 hosts were much more likely to complete and survive the event (Fig. 3f, Supplementary Fig. 2b), especially when the internal cell was a p53 KO cell. Interestingly, many of these failed divisions lead to multinucleation (Supplementary Fig. 2b) and subsequent tripolar mitosis (Fig. 3f, Supplementary Movie 9). Notably, tripolar mitosis was only seen in multinucleated cells and was confirmed using tubulin staining to indicate the three centrosomes (Fig. 3g). Remarkably, tripolar mitosis did result in viable daughter cells and daughter cells were themselves seen to undergo a further round of tripolar mitosis (Fig. 3f, Supplementary Movie 9). These results prompted us to test the inherent rate of tripolar mitosis in A431 cells. Of all cell divisions, only 1% of the non-engulfing mutant p53 A431 cells divided as a tripolar mitosis and no tripolar mitoses were seen in p53 KO A431 cells (Fig. 3h left). Of all dividing host A431 cells, that had a cell internalised for at least 60 min prior to that disrupted a previous division, 32% of mutant p53 host cells compared to 5% of p53 null host cells underwent tripolar mitosis (Fig. 3h right). This suggests that tripolar mitosis is caused by engulfment and not just related to mutant p53 expression. Interestingly, we also observed tripolar mitosis in mutant p53 patient tumours and cell lines that had particularly high numbers of CIC structures (Supplementary Fig. 2c, d). Together, these data suggest that the increased numbers of CIC structures are correlated to an increased ability of cells to survive cell division after entotic engulfment, which was mostly observed in mutant p53 cells that had engulfed p53 null cells.

### CIC in mice is associated with enhanced tumour growth

**Fig. 4:**
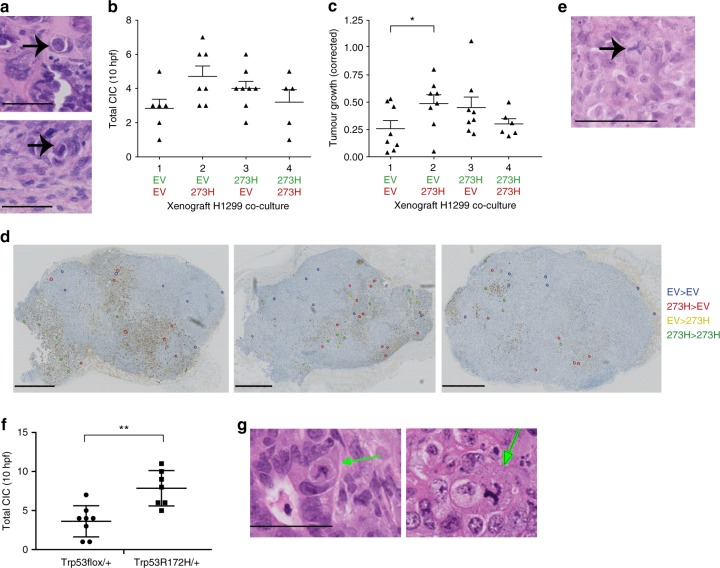
Increased CIC activity in mutant p53 xenografts is pro-tumorigenic. **a** Example images of the CIC structures that were observed in H&E stained sections of mice xenograft tumours, following dissection and fixing. Scale bars = 50 μm. **b** Results of CIC quantification for xenograft tumours of four different H1299 co-cultures (as previously described in Fig. 1). Error bars indicate SD of three hpf of eight mice per group. **c** Dot plot representing corrected tumour growth (tumour size/number of days for the tumour to grow to its max size) for the same tumours and mice as in **b**. Error bars indicate SD of eight mice per group. *p < 0.05. **d** p53 stained xenograft sections from three different mice from group 2 (EV/mCherry with R273H/GFP co-cultures). As indicated with coloured rings, different combinations of CIC involving mutant (273H) and non-mutant (EV) cells were observed including 273H engulfing 273H (green), 273H engulfing EV (red), EV engulfing EV (blue), and EV engulfing 273H (yellow). Scale bars = 2.5 mm. **e** An example of a tripolar mitosis, which was often observed in H&E stained xenograft tumour sections. Scale bar = 100 μm. **f** Quantification of CIC structures in H&E stained slides from pancreatic tumours of Pdx1-Cre; LSL-^KrasG12D/+^; Trp53^flox/+^, and Pdx1-Cre; LSL-^KrasG12D/+^; LSL-Trp53^R172H/+^mice. **g** H&E stained sections from pancreatic tumours of Pdx1-Cre; LSL-^KrasG12D/+^; LSL-Trp53^R172H/+^ mice. Arrows indicate CIC and the scale bar = 50 μm

Previous studies involving CIC often showed an association between CIC and tumour aggressiveness^8–13^, but to our knowledge, the effects of CIC on tumour growth have not been characterized in a carefully controlled animal study. We therefore hypothesized enhanced growth of tumours with increased CIC. Our H1299 cell model of lung adenocarcinoma with and without mutant p53 in combinations similar to those in Fig. 1, provided the ideal model to test this. We injected eight nude mice with the four different H1299 co-culture mixtures (EV or R273H with GFP or mCherry) and monitored tumour progression. We measured tumour size, CIC structures and p53 status and identified CIC structures within the tumours (Fig. 4a, b) to similar extents as our tissue culture experiments (Fig. 1c) showing that cells retain their CIC formation ability in vivo. Most interestingly, tumours with the highest CIC numbers also had the largest average tumour size (Fig. 4c). Previous studies implicate mutant p53 expression with increased proliferation, decreased cell death, and tumour growth^36,37^. However, in agreement with our previously published H1299 xenograft studies^31^, homogenous mutant p53 expression in H1299 cells did not impact on the ability of the primary xenograft tumours to grow (co-culture group 1 compared to group 4, Fig. 4b, c). H1299 mutant p53 and EV control cells were thoroughly tested for cell cycle progression and did not show differences in the incorporation of EdU (Supplementary Fig. 3a), suggesting that just mutant p53 in our H1299 cells does not promote proliferation or tumour growth. These data therefore suggest that the increased growth we observed in co-culture groups 2 and 3 is related to the increased ability of mutant p53 cells to survive engulfment and not an effect of mutant p53 on proliferation. Notably, co-culture group 3 results were slightly lower than group 2, which could possibly be explained by a lower number of p53 expressing cells (Supplementary Fig. 3b). Most CIC structures were detected on the borders of p53 positive and p53 negative areas of cells (Fig. 4d), which suggests a preference for CIC formation to take place in more heterogeneous populations, such as seen in our mutant p53/p53 null co-cultures (Fig. 1). Notably, tripolar mitoses were also observed in these xenograft tumours (Fig. 4e).

To asses whether CIC structures occur in genetically engineered mouse models of p53, we turned our attention to the KPC mice in which mutant p53 R172H (equivalent to human R175H) is expressed in combination with a K-Ras12D activating mutation under a pdx1 promoter confining expression to the pancreas. Tumours of mutant p53 Pdx1-Cre; LSL-^KrasG12D/+^; LSL-Trp53^R172H/+^ mice are highly metastatic compared to Pdx1-Cre; LSL-^KrasG12D/+^; Trp53^flox/+^mice^38^ that are known to invariantly undergo LOH of p53 and are therefore considered to be p53 null. CIC structures were detected more frequently in the pancreatic tumours of mutant p53 but not in the p53 null mice (Fig. 4f, g)^38^. Interestingly, tripolar mitoses in CIC structures were often observed in the mutant p53 mice and not in the p53 null mice (Fig. 4g, right hand panel).

Together, these data confirm that entotic engulfment is related to p53 mutation status and suggest a pro-tumorigenic function for the engulfment event.

### CIC in lung adenocarcinoma is related to poor outcome

**Fig. 5:**
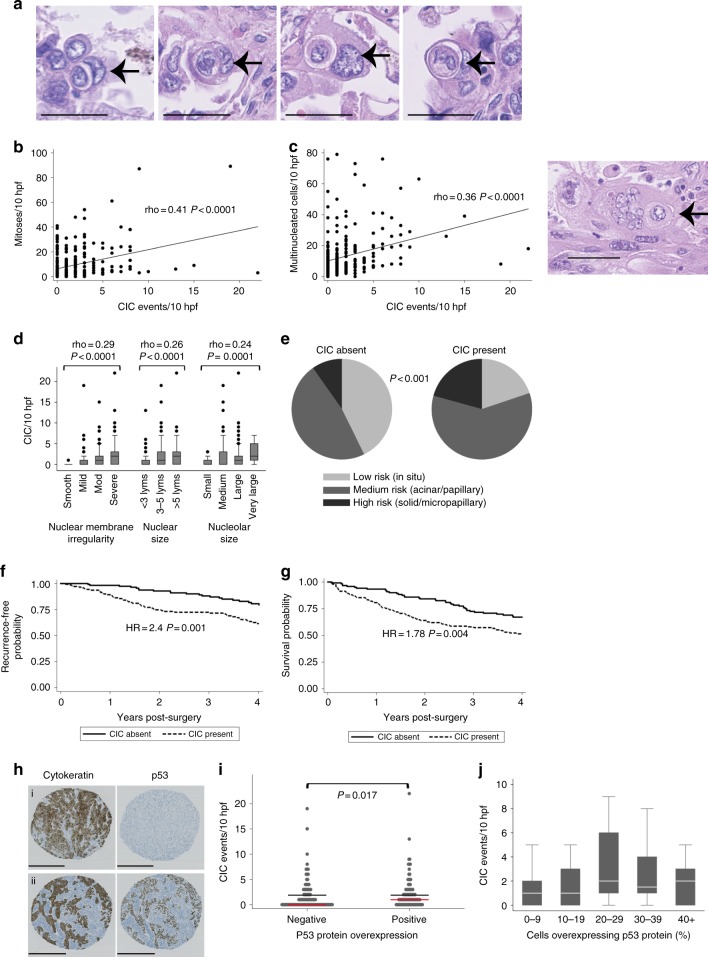
CIC structures in lung adenocarcinoma and correlation with p53 status. **a** H&E stained sections of lung adenocarcinoma containing examples of CIC structures, as indicated by arrows. Scale bars = 50 μm. **b** Correlation between CIC occurrence and mitoses *n* = 273 (Spearman’s ρ). **c** Correlation between CIC occurance and multinucleation *n* = 273 (Spearman’s ρ) (left). H&E stained sections showing multinucleation. Scale bars = 50 μm (right). **d** Correlations between tumour cell nuclear grade (membrane irregularity, nuclear size, and nucleolar size) and CIC. *n* = 273 (Spearman’s ρ). Error bars indicate SEM. **e** Pie charts illustrating the predominant histological growth patterns in cases where CIC is present or absent. High-risk invasive patterns are solid/micropapillary, medium risk patterns are acinar/papillary appearance, and predominantly in situ disease is relatively low-risk disease. **f**, **g** Kaplan–Meier plots comparing disease recurrence (**f**) and all-cause survival (**g**) in patients with tumours with and without CIC. Hazard ratios and *p*-values are obtained from univariate Cox models. **h** Example images of 1 mm cores of lung adenocarcinoma used to construct TMAs and perform p53 expression analysis. A p53 negative case (top panels) and p53 positive case (bottom panels) are shown. Scale bars = 500 μm. **i** Correlation between CIC and p53 mutation status (Wilcoxon rank-sum test *p* = 0.017) (*n* = 209). **j** CIC events plotted per % of cells expressing p53 to show heterogeneity in p53 protein overexpression (outliers omitted for clarity)

We next investigated a role for CIC in correlation with mutant p53 status in patient samples. Mutation of p53 is the most ubiquitous driving genomic event in pulmonary adenocarcinoma^19^, and is highly pleomorphic in its effects upon cellular biology^23^. We therefore first established a cohort of 273 resected surgical lung adenocarcinomas (Supplementary Table 1) and counted CIC events (Fig. 5a) in whole tumour sections. 45% showed CIC and 55% did not. CIC events are highly significantly associated with mitoses (*p* < 0.0001) and the presence of multinucleation/giant cells (*p* < 0.0001) (Fig. 5b, c left). Multinucleated cells were often seen to be engulfing other cells, further suggesting a possible causative link between entotic engulfment and multinucleation (Fig. 5c right). There are also highly significant associations with measures of nuclear grade including nuclear membrane irregularity, (*p* < 0.0001), nuclear size (*p* < 0.0001) and nucleolar size (*p* < 0.0001) (Fig. 5d) and with high-risk histological patterns of disease (Fig. 5e). Furthermore, tumours with observable CIC are strongly associated with earlier recurrence (Fig. 5f) and death (Fig. 5g). This association was preserved in a multivariate Cox model of tumour recurrence (HR = 1.98, *p* = 0.025) including known major predictors of early relapse, indicating statistical independence (Supplementary Table 2). Together, these findings show that CIC structures are common in lung adenocarcinoma, and are associated with tumour grade, virulence, and poor outcome. Using tissue microarrays (TMAs) constructed from our cohort we applied an immunohistochemical assay for p53 protein overexpression (Fig. 5h), which is an indicator of oncogenic activating mutations^39^. A cutoff was optimized using a subset of 36 tumours with known p53 mutation status in order to maximise sensitivity and specificity of mutation detection (90% and 84% respectively). There was a significant relationship between mutant p53 expression and the occurrence of CIC (Fig. 5i). Furthermore, on closer examination we also observed that CIC occurrences peak when numbers of mutant p53-positive cells are intermediate (Fig. 5j). This implies that at very low or very high proportions of mutant p53 positivity, CIC events are relatively rare, suggesting that p53 heterogeneity is linked to higher rates of cellular engulfment, supporting our data in heterogenous cell populations in vitro (Fig. 1) and our mice data (Fig. 4d).

### CIC is associated with DNA damage and genomic instability

**Fig. 6:**
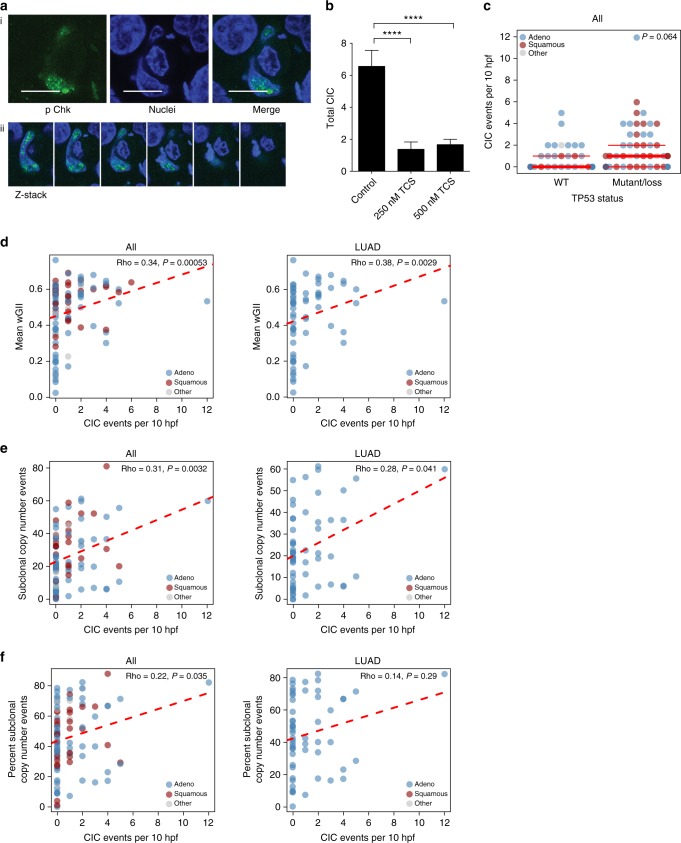
CIC correlates with replicative stress and measures of both genomic instability and sub clonal copy number loads in patients. **a** Z-stack images of non-fluorescent 273H mutant p53 A431 cells that were stained for pChk1 using immune fluorescence,and imaged with confocal microscopy. Scale bars = 20 μm. **b** Quantification of total CIC structures observed in A431 Ctr 273H/mCherry and KO 273H/GFP co-cultures with and without TCS2312 treatment for 24 h. Each bar represents +/−SEM of triplicate experiments ****p < 0.0001. **c** Association between p53 mutations and CIC using the first 100 patients used in the TRACERx study. **d**–**f** Associations between CIC and indicators of genomic instability in all the first 100 lung cancer patient tumours of the TRACERx study (ALL) and or lung adenocarcinoma tumours (LUAD) specifically (*n* = 61). **d** Association between CIC and weighted genome integrity index (wGII) for ALL (left) and LUAD (right). **e** Association between CIC and genomic alterations using sub clonal copy number loads in NSCLC for ALL (left) and LUAD (right). **f** Association between CIC and genomic alterations using percent sub clonal copy number loads in NSCLC for ALL (left) and LUAD (right). Statistical analysis was Spearman’s rank correlation, with rho and *p* values indicated on the graphs

The observation of aberrant mitoses in p53 mutant host cells suggests a link between p53 mutation, cell engulfment, and cell division checkpoint escape. Several checkpoint proteins are involved in maintaining chromosome integrity during cell division. One of these is p-Chk1, which is recruited to the DNA to signal replication stress and has previously been shown to be regulated by mutant p53^40^. On several occasions, we could specifically detect p-Chk1 on the DNA of engulfing A431 cells (Fig. 6a), but never in the normal cell population grown under the same conditions. These data suggest that engulfment causes replication stress that is more detrimental to p53 null cells than it is to mutant p53 cells. To prove that replication stress survival is dependent on mutant p53 induced Chk1 activation, we blocked phosphorylation using TCS2312^41,42^. TCS2312 treatment indeed led to a reduction in the number of CICs detected in mutant p53 cells (Fig. 6b).

Given these associations between CIC, aberrant mitosis and replication stress, we sought evidence that CIC is related to chromosomal abnormalities in human tumour tissue. For this purpose, we quantified CIC in digital images of tumour sections from the TRACERx cohort, examining a set of 100 human non-small cell lung cancers^43^. These tumours had previously been subjected to multiregion whole-exome sequencing, enabling quantification of somatic single nucleotide and copy number variants, as well as measures of genomic intratumour heterogeneity. We identified a trend (*p* = 0.064) towards increased frequency of CIC structures in patients with p53 mutations (Fig. 6c). Furthermore, CIC was highly significantly associated with structural chromosomal changes as measured by weighted genome integrity index (wGII) when considering all patients (ALL) (Fig. 6d left). This association was slightly stronger in lung adenocarcinomas (LUAD) (Fig. 6d right). Crucially there was no relationship with single nucleotide variants (Supplementary Fig. 4a), suggesting that any causal link between CIC and genomic alteration is related to chromatin structural aberration rather than sequence changes. Furthermore, while CIC was not associated with the amount of clonal copy number alterations (Supplementary Fig. 4b), a significant association was found between CIC and both the overall amount of subclonal copy number alterations (Fig. 6e), and the percent subclonal copy number alterations (Fig. 6f).

## Discussion

Although CIC structures have been described in histological sections of solid tumours for decades^9,18^, we still know remarkably little about their physiological or clinical relevance. In this work, we have shown highly significant relationships between CIC and patient outcome as well as architectural and cytological measures of tumour grade. We suggest that quantification of CIC may be a useful component of prognostic lung cancer grading systems, which are nearing routine clinical implementation. CIC therefore merits further investigation as a prognostic biomarker in this regard.

The origin of multinucleate cells in tumours is unclear, with cell fusion and failure of cytokinesis in mitosis both being possible^44,45^. In our system, the associations between CIC and multinuclearity, in human tumours, and the observed link between CIC and multinucleation in cell culture, strongly supports a model in which entotic engulfment induces multinuclearity by interfering with cytokinesis.

We detected a highly significant association between CIC event frequency and mutant p53 expression. Although CIC ascribed to entosis has been related to molecular markers such as E-Cadherin and K-Ras in tissue culture^46^, no association with specific genomic changes has previously been demonstrated. Crucially we show in cell culture that mutant p53 expression is sufficient to greatly enhance the cellular engulfment process which leads to CIC structures, strongly supporting a causative mechanistic link between p53 mutation and CIC in tumour tissue. Our results raise the question as to what mechanism(s) underlie entotic engulfment. This is likely to be complex, given the numerous receptor-triggered pathways leading to cell engulfment in classical phagocytosis^47^. However, in line with our previous work on mutant p53 function in invasion and metastasis, we have provided evidence that EGFR and alpha5/beta1 integrin and ROCK signaling are involved. Future studies will employ screens to identify whether other receptors and cell signaling molecules are involved.

**Fig. 7:**
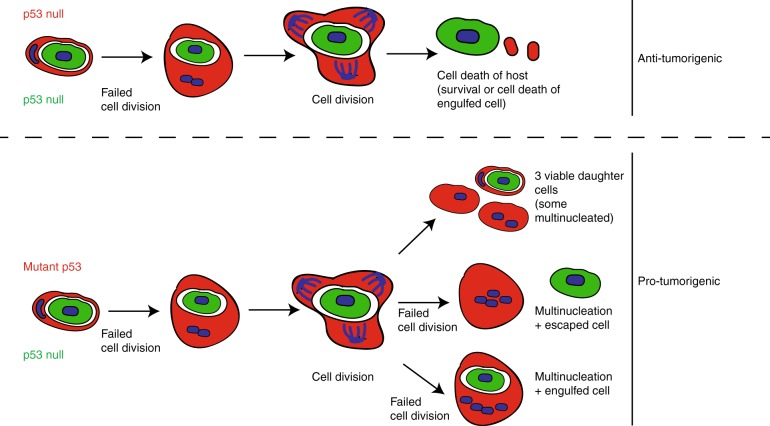
Schematic model of the consequences of cell engulfment in mutant p53 and p53 null cells. In p53 null cells, engulfment followed by a failed cell division, multinucleation, and subsequent cell division most often leads to cell death. Mutant p53 cells that divide following engulfment, failed division, and multinucleation experience aberrant mitoses, leading to the generation of three daughter cells via tripolar mitoses and further multinucleation

The associations between CIC and both patient outcome and tumour grade show that cellular entotic engulfment is an indicator of tumour virulence. We found that p53 null cells often die after engulfing other cells. In contrast, mutant p53 cells which engulf others and in particular p53 null cells generally survive, and often go on to survive failed mitotic events resulting in multinucleation and tripolar mitotic events (Fig. 7). p53 has well-described roles in ensuring normal segregation of chromosomes at mitosis, being involved in normal centrosome clustering^40^ and in the operation of mitotic checkpoint controls^48^. Conversely, mutant oncogenic p53 function is permissive of abnormal mitotic events, and permits passage through mitotic checkpoints that would otherwise induce apoptotic cell death of abnormal cells, causing chromosomal changes^49,50^.

We show that mutant p53 activated p-Chk1 to survive replication stress in cells with genomic abnormalities^40^. Inhibition of p-Chk1 by others was found most useful in inhibiting growth of mutant p53 positive tumour xenografts^51,52^. In addition to this, engulfment has previously been shown to favour aneuploidy in subsequent mitoses due to steric interference with the normal dynamics of cell division by the ingested cell^53^. This was accompanied by aberrant gene copy numbers in host upon entosis^53^. Therefore the combination of cell ingestion and mutant p53 status might be expected to drive abnormal mitotic events; the entotic engulfment event would cause abnormal cellular division, and p53 mutation would allow the abnormal progeny to survive. This is what we observe in cell culture.

Crucially, when we sought associations between CIC, p53 and large-scale genomic aberrations in a set of tumours for which we also held full genomic characterization, we observed the predicted association between CIC and somatic copy number aberrations, but found no association with single nucleotide variants, consistent with a model in which CIC affects chromosome integrity/segregation but not the fidelity of DNA replication. Interestingly, chromosomal instability has previously been reported in the Pdx1-Cre; LSL-^KrasG12D/+^; LSL-Trp53^R172H/+^ mice and not the Pdx1-Cre; LSL-^KrasG12D/+^^38^, further supporting the idea that mutant p53 expression and CIC are associated with genomic instability.

Although most reports link CIC to pro-tumorigenic effects, some studies suggest anti-tumorigenic or anti-metastatic properties for CIC^7,11^. Our study provides a possible explanation for this discrepancy based on p53 status; CIC in mutant p53 cells drives aneuploidy, as described above, but in cells with no or wild-type p53, the host cells more frequently die (Fig. 7).

Our results also suggest that CIC occurrence is higher in heterogeneous populations of cancer cells. Intratumour heterogeneity is a crucial issue in cancer diagnosis and treatment, with the potential for treatment-resistant subclones resulting in disease relapse. The heterogeneity of the lung cancer has recently been demonstrated, and at least 80% of cases are detectably polyclonal^54^ and subclonality of TP53 mutations in particular have been described^43^. In keeping with this, in our IHC assay of p53 protein expression, the numbers of positively stained nuclei varied widely from case to case suggesting that in some cases p53 mutation was subclonal (Fig. 5h). Besides heterogeneity due to genomic clonality, p53 protein expression is a highly responsive to external stress stimuli, which might also underlie the variation in p53 IHC staining that we and others can see within one tumour. Of particular interest for this study is the finding that mechanical stress can induce p53 expression^25^. When analyzing CIC structures in different cell lines (Supplementary Fig. 1k), we noticed that the cell density can greatly affect CIC numbers with higher densities often leading to more CIC. Hypothetically, mutant p53 expression mediated regulation of CIC could therefore provide a mechanism in crowded tumours to create space for the most aggressive cells to survive.

Our observations of more frequent CIC occurrences in p53-heterogeneic populations, in patients, in tissue culture and in xenografts, support our model in which mutant p53 and CIC synergise to cause genomic heterogeneity via chromosome destabilization. Excitingly, this relationship might also be evidence of direct clonal competition, with CIC events representing a mechanism of interclonal conflict. Understanding the mechanisms underlying CIC is therefore crucial, as inhibition of CIC might both stabilize cancer cell genomes and reduce the scope for successful interclonal competition and natural selection during cancer therapy.

## Methods

### Archival tissues and morphological scoring

Patient samples were obtained from the Leicester thoracic tumour archive, a single-centre retrospective cohort study of surgical lung cancer specimens, with ethical approval from the NRES East Midlands Research Committee (REC reference 14/EM/1159). The set included in this study contains 994 surgical specimens from primary pulmonary adenocarcinoma surgeries performed with curative intent in Leicester between 1998 and 2014. Full clinicopathological data, were recorded for each patient. For this study, we included 273 of the 994 patients that were first readily available and complete in terms of full tumour sections, images and patient data, and the cohort is summarised in Supplementary Table 1.

In brief, diagnostic slides of tumour tissue were recovered from the archive and digitized (at 40× magnification) using a Hamatsu Nanozoomer-XR C12000 scanner (Hamamatsu Photonics Ltd, UK). Ten high power fields (hpf) of the scanned images (equivalent to 2.4 mm^2^) were examined and scored for; mitotic cells, multinucleated cells, nuclear membrane irregularity (smooth/mild/moderate/severe), nuclear size (median diameter < 3×/3−5×/>5× lymphocyte diameter), nucleolar size (small/medium/large/very large) and also predominant disease growth pattern (low risk, in situ/medium risk, acinar or papillary/high risk, solid or micropapillary) using H&E staining. For scoring of CIC structures, 10 hpf areas within epithelial-rich viable malignancy on digital images of tumours were analysed using NDPView2 software. CIC occurrences were counted within these areas. To count as a CIC structure, at least four of the six following features were required to be unequivocally identifiable: nucleus of internalised cell, cytoplasm of internalized cell, nucleus of engulfing cell, cytoplasm of engulfing cell, ‘moon-shape’ host nucleus, and intervening vacuolar space. Only events where 100% cell internalisation was evident were counted.

### TRACERx

TRACERx is a prospective cohort study in which primary tumours collected from patients with stages I-IIA non-small cell lung cancer undergoing curative resection are subjected to multiregion whole-exome sequencing^43^. The study (Clinicaltrials.gov no: NCT01888601) is sponsored by University College London (UCL/12/0279) and approved by an independent Research Ethics Committee (13/LO/1546). TRACER is funded by Cancer Research UK (grant number C11496/A17786) and coordinated through the Cancer Research UK & UCL Cancer Trials Centre.

The following eligibility criteria were used to recruit patients into the TRACERx study:

Inclusion criteria: Written Informed consent, Patients ≥18 years of age, with early stage I-IIIA disease who are eligible for primary surgery, Histopathologically confirmed NSCLC, or a strong suspicion of cancer on lung imaging necessitating surgery (e.g., diagnosis determined from frozen section in theatre), primary surgery in keeping with NICE (National Institute for Health and Care Excellence) guidelines planned, agreement to be followed up in a specialist centre, ECOG performance status 0 or 1. Exclusion criteria: Any other current malignancy or malignancy diagnosed or relapsed within the past 5 years (other than non-melanomatous skin cancer, stage 0 melanoma in situ, and in situ cervical cancer), psychological condition that would preclude informed consent, treatment with neo-adjuvant therapy for current lung malignancy deemed necessary, adjuvant therapy other than platinum-based chemotherapy and/or radiotherapy, known human immunodeficiency virus (HIV), hepatitis B virus (HBV), hepatitis C virus (HCV) or syphilis infection, sufficient tissue, i.e., a minimum of two tumour regions, is unlikely to be obtained for the study based on pre-operative imaging.

Clonal and subclonal copy number alterations and mutations were determined as described^43^, wGII scores^55^ were determined independently for each tumour region, and averaged across all regions. Association between CIC and genomic alterations were determined using Spearman Rank Correlation. *TP53* was considered altered if harbouring a known cancer driver mutation, as described^43^. Association between CIC and *TP53* status was assessed using a Wilcoxon Rank Test. All sequencing data have been deposited in the European Genome–Phenome Archive under accession number EGAS00001002247.

### Animal models

The project license was granted under the Animals (Scientific Procedures) Act 1986. Four groups with eight healthy male mice in each were subcutaneously injected with cell xenografts. These numbers were predicted to render significant results on tumour growth based on pilot experiments as well as previous experiments in which we examined tumour growth of H1299 cells^31^. The mice were 40+-day-old balb/c nude/male mice (Jackson laboratories), housed at the Division of Biomedical Services at the University of Leicester in individually vented cages enriched with tissues and cardboard shelter. Mice were kept on a standard day/night cycle at ambient room temperature and chow and drinking water accessible ad libitum. Bias was reduced by random allocation of mice to the four groups, and blind analysis for the duration of the experiment. Briefly, 100 μl of cell suspension (containing 5 × 10^5^ H1299 cells in four different combinations) was combined with 100 μl of Matrigel (356231 Corning). Mice were anaesthetised in the morning before noon using isofluorane (flowmeter 500/1000 ml/min) as a rapid induction and recovery anaesthetic with the vaporizer set to 5% until signs of recumbent. Mice were then placed on a nosecone with the vaporizer set to 1.5% isofluorane before mice were injected with cells in a sterile hood and placed directly back into a clean standard housing cage and monitored for the rest of the day. Subsequently, mice were monitored daily for weight changes, signs of distress or infection and tumour growth using caliper measurements. We did not note >10% weight loss, infection or any other adverse events throughout our study. Mice were culled (using a schedule 1 approved technique of cervical vertebrae dislocation) when the tumour measurement reached a maximum size of 1200 mm^3^ as stipulated in our project license. Following dissection and checks for local and distant metastasis, excised tumours were fixed in formaldehyde and sections were cut. Sections were then stained according to standard H&E staining protocols and scored for CIC, as previously described in FFPE patient samples.

H&E stained slides from pancreatic tumours of Pdx1-Cre; LSL-^KrasG12D/+^; Trp53^flox/+^ and Pdx1-Cre; LSL-^KrasG12D/+^; LSL-Trp53^R172H/+^mice were obtained from Dr Morton (described in ref. ^56^) and were scanned using the Leica SCN 400. CIC structures were quantified as described before.

### Tumour DNA extraction and p53 sequencing

Thirty-six 1 mm cores of lung adenocarcinoma tumour were removed from the FFPE adenocarcinoma blocks. Excess paraffin was removed and remaining tumour placed into a micro centrifuge tube. DNA was then extracted via the GeneRead DNA FFPE kit (Qiagen, UK) according to manufactures instructions. Once extracted, DNA was then sequenced using Ion Torrent Next Generation Sequencing (NGS) technology (Ion AmpliSeq^TM^ Colon and Lung Cancer Panel) gain-of function p53 mutations were identified by point mutations in the DNA binding region (Supplementary Table 3).

### Tissue micro-arrays and p53 immunohistochemistry

TMAs were constructed with three 1 mm cores of lung adenocarcinoma tumour per patient case, with 60 cases on each slide. Two consecutive TMA sections were stained for p53 (Dako DO-7) and cytokeratin AE1/AE3 (Dako) using an Agilent™ Link 48 instrument following antigen retrieval in a PT Link unit, following manufacturer’s recommendations. Counterstained slides were then scanned using a Nanozoom XR C12000 digital slide scanner and analysed with Visiopharm analysis software applications. Briefly, p53 and cytokeratin stained cores were aligned and, nuclei within cytokeratin-positive areas were identified. P53-positive nuclei were identified by the application of a manually optimized threshold, which counted strongly staining nuclei, and the proportion of positive nuclei was calculated as a percentage. An average for the three cores per case was calculated. Mutational status from the 36 cases in which this data was obtainable was used for receiver operator curve (ROC) analysis to set a cutpoint to binarise the mean nuclear positivity variable. The area under the ROC curve was 92%, and the optimal cutpoint (1.9% positive cells) was identified by the Liu method. This cutpoint yielded a sensitivity of 90% and specificity of 84%.

For xenograft tumours, a different p53 antibody (1:1000 Dako, Denmark) was used to perform immunohistochemistry and this was done with an AutostainerLink48 (Dako).

### Cell culture and constructs

A431, A549, U2OS, BxPC-3, and H1299 cell lines were obtained from ATCC. PC-9, H322m, H358, and H23 cell lines were a kind gift from Dr. Howard Pringle. HCT116 cells were a gift from Dr Bert Vogelstein. Cell lines were maintained in Dulbecco’s modified Eagle’s medium (Gibco, UK) supplemented with 10% foetal bovine serum (Sigma, UK) and 1% pen/strep (Gibco, UK) at 37 °C with 5% CO_2_. Cells were regularly checked against mycoplasma. H1299 cells expressing mutant p53 have been established before^31^ and were stably transfected with eGFP and mCherry (Clontech, France) and selected using stringent FACS sorting (BD Aria III). A431 p53 knockout cells were made with a CRISPR construct pLV-U6g-EPCG with target sequence TCCATTGCTTGGGACGGCAAGG (Sigma, USA) according to the manufacturer’s protocols and tested for p53 expression using western blot, where after they were stably transfected with eGFP or mCherry. p53 22/23, 175H, and 273H constructs were described in ref. ^31^. R248W mutant p53 was generated using mutagenesis with the following oligos: fw- TGCATGGGCGGCATGAACCaGAGGCCCATCCTCACCATC, rev- GATGGTGAGGATGGGCCTCTGGTTCATGCCGCCCATGCA.

### CIC quantification (high content screening)

eGFP and mCherry cells were seeded together in 24-well plates at equal density and left for 48–120 h depending on the cell line. Before imaging, media was removed, nuclei stained with 10 μM Hoechst (Molecular Probes, Life Technologies, UK) for 20 min and fresh media added. Imaging of living cells was done with cellomics high content imaging (ThermoFisher Scientific, UK) or the Operetta CLS (Perkin Elmer) at 20× magnification. Twenty-five 20× fields were then analysed for each well to score for red and green cell engulfment events. If one cell was more than 75% enclosed within another cell, then this was marked as an engulfment event. Red engulfing red and green engulfing green were not counted as these cannot always be confidently detected and classed as background engulfment. Cells were incubated with 1 ng/ml EGF (Sigma), 0.5 μg/ml mAb16 (BD Biosciences), 1 or 5 μM Gefetinib (Sigma), 250 or 500 nM TCS2312 (Tocris), 5 μM Y-27632 (Merck- Millipore) one day after seeding until imaging. Plates were coated in 1 μg/cm^2^ fibronectin (Sigma), 2 μg/cm^2^ collagen (Sigma) or 1 μg/cm^2^ gelatin (Sigma).

For transient transfections, A431 p53 KO cells were transfected with a combination of mCherry (0.2 μg) with the indicated p53 constructs or a pcb6 control (1 μg). In pilot experiments, we determined that >80% of cells that had incorporated mCherry, also incorporated p53. Engulfment was quantified based on the number of red cells engulfing neighbours as a percentage of all red cells using Hoechst as labeling to indicate individual cells. A minimum of 750 cells per experiment were counted.

For non-fluorescent cells, cells were stained in 4 nM Calcein for 1 h, washed and stained with 10 μM Hoechst for 20 min. Twenty-five 20× fields were analysed for the typical engulfment features. Engulfment was further confirmed with Calcein staining. The percentage engufment was corrected for the total number of cells detected with the Harmony software in each field.

### Time lapse analysis

For time-lapse experiments, imaging was started 24–72 h (depending on the cell line) after seeding cells in a 35 mm glass bottom dish (Mattek, USA) with images taken every 10 min. Imaging was carried out by a Nikon biostation IMQ microscope (Nikon, UK), with live brightfield and fluorescent imaging capabilities. For time-lapse quantification of A431 CIC outcomes, imaging was started 24 h following cell seeding. Random fields were selected to determine internalization. For outcome experiments, CIC structures were located and imaged for 72 h, in both phase contrast and fluorescent channels, to determine outcome for both host and internal cells.

### Immunofluorescence and confocal imaging

Fluorescent cells were co-cultured on glass coverslips for 48–120 h, before being washed 3× in PBS and fixed with 4% paraformaldehyde (Thermo Scientific, USA) for 10 min at 4 °C. Cells were then washed and coverslips mounted on slides with vectashield mounting medium with DAPI (Vector Laboratories, USA) and imaged on a confocal microscope (Zeiss LSM 510 META NLO, Carl Zeiss, Germany). Z-stacks were generated using optimal intervals.

Non-fluorescent cells were washed after fixation as above with 3× PBS, permeabilized with 0.5% Triton X-100 in PBS for 1 min, washed 3× PBS and blocked in 1% bovine serum albumin (BSA) in PBS for 30 min. Cells were then incubated with primary antibodies (1% BSA/PBS) for 1 h. Antibodies used were; p53 (1:200, Santa Cruz), mCherry (1:200, Abcam), Integrin (1:100, Abcam), mAb16 (1:100 BD Biosciences), Tubulin (1:200, Abcam), p-Chk-1 (1:100 Cell signaling), E-Cadherin (1:2000, Cell signaling), β-Catenin (1:2000 Abcam). Cells were washed in PBS (3×), and incubated in Alexa 488, 594 or 633 conjugated secondary antibodies (1:250, Invitrogen) before mounting. For pHrodo (1 μg/ml, Invitrogen) or CalceinAM (1:2000 Invitrogen, Molecular Pobes, USA), living cell were stained for 1 h before washing (2× PBS). Calcein AM stained cells were imaged straight away while pHrodo labeled cells were imaged 24 h later. Fluorescent intensity of E-Cadherin and β-Catenin was determined using Plot Profile in Fiji on the average intensity of lines. Imaris was used to create a 3D structure from a Z-stack made on the confocal microscope.

### Western blot

A431 ctr or KO cells were lysed on ice in NP-40 lysis buffer (100 mM NaCl 100 mM Tris pH8, 1% NP-40) for 15 min. Pellets were discarded after spinning at max speed for 15 min at 4 °C and supernatant was separated in SDS-PAGE and transferred onto nitrocellulose membrane. After blocking with 5% skimmed milk (Marvel, Camlab, Cambridge, UK) in TBS (Santa Cruz)/0.1% Tween20 (Sigma) for 30 min at RT, the membranes were incubated with primary antibodies in 5% skimmed milk/TBS-T. The following primary antibodies were used for western blotting: EGFR (Cell Signaling), pEGFR (Abcam), Actin (Millipore).The blots were washed three times with TBS-T and then incubated with corresponding IRDye secondary antibodies 680RD or 800CW (Li-Cor, Cambridge, UK, 0.05 µg/ml). After three times washing with TBS-T protein signals were visualized using the ODYSSEY Sa infrared system (Li-Cor) and Image Studio software 2.0 (Li-Cor). Full scans of Western blots are shown in Supplementary Figure 5.

### Cell proliferation rate measurement

EV and mutant p53 H1299 cell line proliferation rates were measured on a flow cytometer following staining with 5-ethynyl-2′-deoxyuridine (Edu). This was done with a Click-iT EdU Flow Cytometry Assay Kit (Invitrogen, Molecular Probes), according to manufacturers’ instructions. Results were analysed using BD FACS Diva software (version 8.0.1).

### Statistics

In the cell culture experiments, statistical comparisons between groups were made using a non-parametric *T* test.

For histopathological data and mice xenographs, differences between two unpaired groups were detected using the non-parametric Mann–Whitney test, and between multiple groups using the non-parametric Kruskal–Wallis test. Associations between paired variables were quantified using Spearman’s rank correlation. Survival models were constructed using the Cox method. Data were modeled over a 4-year period, and radiological evidence of recurrence was taken as the endpoint. The proportional hazards assumption was tested by inspection of log–log plots. Survival plots were prepared using the Kaplan–Meier method. Prism 6 or 7 (Graphpad) and Stata SE 13 were used for all analyses.

### Data availability

Authors can be contacted to provide raw data upon reasonable request.

## Electronic supplementary material

Supplementary Information

Description of Additional Supplementary Files

Supplementary Movie 1

Supplementary Movie 2

Supplementary Movie 3

Supplementary Movie 4

Supplementary Movie 5

Supplementary Movie 6

Supplementary Movie 7

Supplementary Movie 8

Supplementary Movie 9
