# Publisher Correction: Genomic instability in mutant p53 cancer cells upon entotic engulfment

**DOI:** 10.1038/s41467-018-06026-2

**Published:** 2018-08-28

**Authors:** Hannah L. Mackay, David Moore, Callum Hall, Nicolai J. Birkbak, Mariam Jamal-Hanjani, Saadia A. Karim, Vinaya M. Phatak, Lucia Piñon, Jennifer P. Morton, Charles Swanton, John Le Quesne, Patricia A. J. Muller

**Affiliations:** 1 MRC Toxicology Unit, Lancaster Road, LE1 9HN Leicester, UK; 2 Cancer Studies, University of Leicester, Leicester, LE1 7RH UK; 3 Cancer Research UK Manchester Institute, The University of Manchester | Alderley Park, SK10 4TG Manchester, UK; 4 Translational Cancer Therapeutics Laboratory, The Francis Crick Institute, 1 Midland Rd, London, NW1 1AT UK; 5 Cancer Research UK Lung Cancer Centre of Excellence, University College London Cancer Institute, Paul O’Gorman Building 72 Huntley Street, London, WC1E 6BT UK; 6 Department of Medical Oncology, University College London Hospitals, 235 Euston Rd, Fitzrovia, London, NW1 2BU UK; 7 CRUK The Beatson Institute, Garscube Estate, Switchback Road, Glasgow, G61 1BD UK; 8 Institute of Cancer Sciences, University of Glasgow, Switchback Road, Glasgow, G61 1BD UK; 9 Department of Histopathology, Glenfield Hospital, University Hospitals Leicester NHS Trust, Groby Road, Leicester, LE3 9QP UK; 10 Present Address: Institute of Cancer and Genomic Sciences, University of Birmingham, Birmingham, B15 2TT UK

Correction to: *Nature Communications* 10.1038/s41467-018-05368-1; published online 03 August 2018

The original version of this article incorrectly omitted an affiliation of Patricia A. J. Muller: ‘Cancer Research UK Manchester Institute, The University of Manchester | Alderley Park, Manchester, SK10 4TG, UK’. This has been corrected in both the PDF and HTML versions of the article.

