# Supplementary material for: Genomic instability in mutant p53 cancer cells upon entotic engulfment
Source: Nat Commun. 2018 Aug 3;9:3070. doi: 10.1038/s41467-018-05368-1 (PMC6076230; doi:10.1038/s41467-018-05368-1)
Supplement: Supplementary file 1 — Supplementary Information [file 41467_2018_5368_MOESM1_ESM.pdf]

Supplementary figure 1.

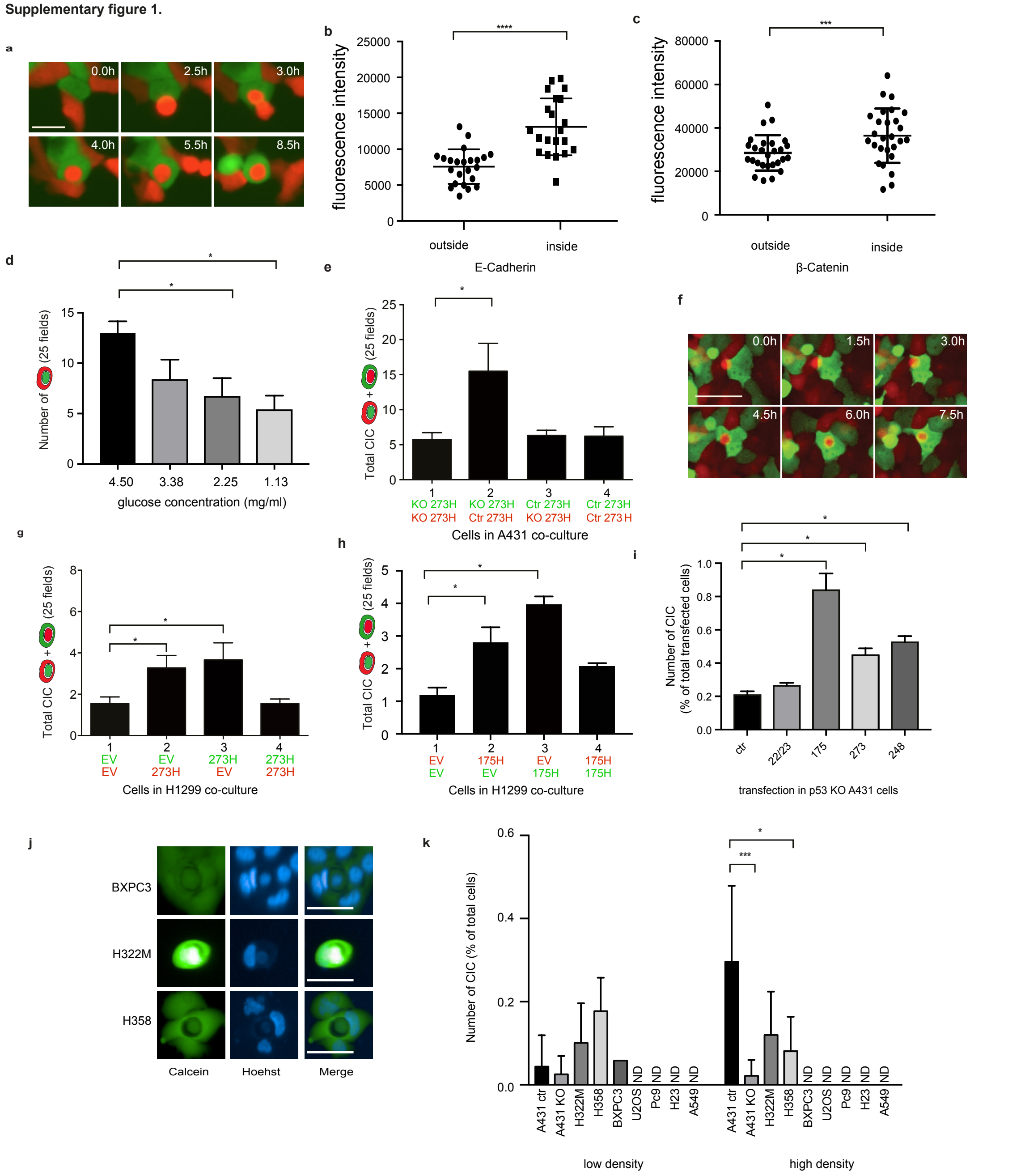

Supplementary Figure 1.
(a) Time-lapse imaging of CIC formation between a 273H/GFP H1299 cell and EV/mCherry H1299 cell. Images were taken every 1.5 hours for 7.5 hours. Scale bar = 50µm. (b and c) Fuorescent intensity staining of E-cadherin (b) or β-Catenin (c) on the membrane between the host cell and the engulfed cell (inside) or between neighbouring cells (outside). (d). Number of CIC events in mutant p53 (green)/ KO p53 (red) co-cultures in which engulfment by red cells was quantified under low glucose levels. (e) Quantification of CIC in A431, as shown in Fig 1(e), but with red and green cell engulfing events added together. (f) Time-lapse imaging of CIC formation between a Ctr 273H/GFP A431 cell and KO 273H/mCherry cell, with imaging over 8.5h. Scale bar = 50µm (g and h) Quantification of CIC in H1299 (EV/ 273H in (g) and 175H in (h)), as detailed in Fig1 (b), but with red and green cell engulfing events added together. Each bar represents +/- SEM of triplicate experiments * p<0.0454. (i) Percentage of CIC in the population of A431 p53 KO cells that were transfected with a mutant (175H, 248W or 273H) or 22/23 p53 construct, compared to mCherry transfected CIC. Each bar represents +/- SEM of triplicate experiments * p=0.0372. (j) Fluorescent microscope images of CIC in BXPC3, H322M and H358 cells stained with Calcein and Hoechst.Scale bars = 50µm (k) Percentage of CIC in A431 ctr, A431 p53 KO, H322M, BXPC3, U2OS, Pc-9, H-23 and A549 cells plated in low or high density. ND= Not Detected. Error bars indicated SEM of three experiments.

Supplementary figure 2.

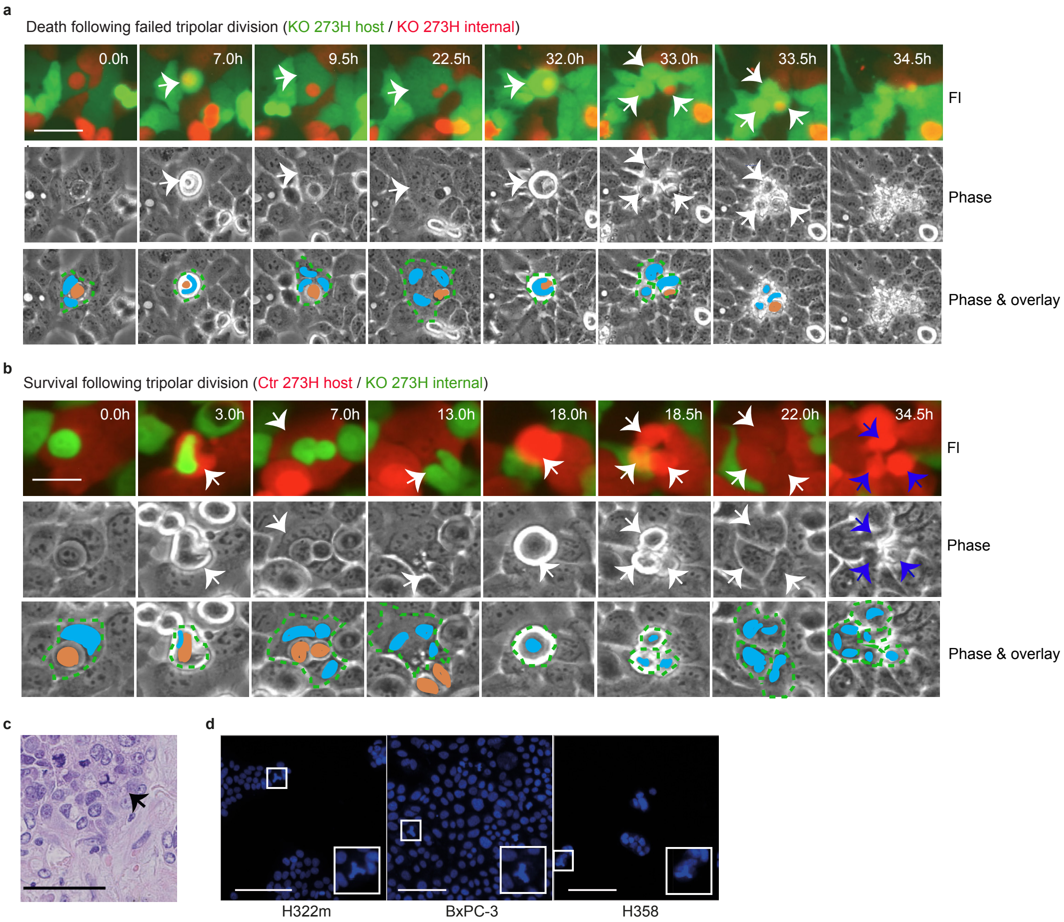

Supplementary figure 2.
(a) Phase contrast time-lapse images following the formation of a multinucleated A431 cell (blue nuclei in the superimposed phase contrast image below seen after 8hrs in this time lapse) following a failed division event, which was due to the presence of an internalised A431 cell.(indicated by an orange nucleus) Scale bar = 20µm. (b) Example of a tripolar mitosis in an H&E stained section of lung adenocarcinoma tumour. Scale bar = 100µm. (c) DAPI stained cell lines, in which we observed high number of CIC structures, where tripolar mitosis events can also be observed. Scale bars = 100µm. (d) CRISPR KO 273H host and (e) Ctr 253H mutant p53 host, both of which engulfed a CRISPR KO 273H internal cell. Orange nuclei indicate engulfed cells and blue nuclei indicate cells that are engulfing.

Supplementary figure 3.

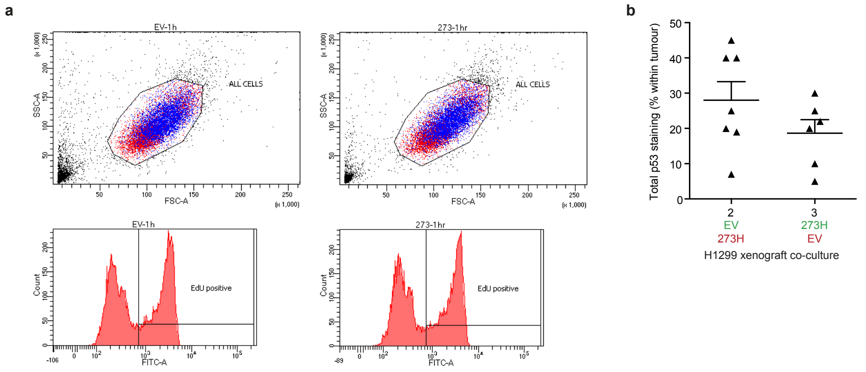

Supplementary figure 3.

(a) EdU staining in ctr (EV) or mutant p53 R273H (273H) H1299 cells. Indicated in the top panels is the scatter plot with EdU positive cells in blue. The lower histograms show the percentage of EdU positive cells (b) Quantification of percentage of p53 positive tumour cells per area in heterogeneous xenograft co-culture groups 2 and 3. Each bar represents +/- SD of eight animals.

Supplementary figure 4.

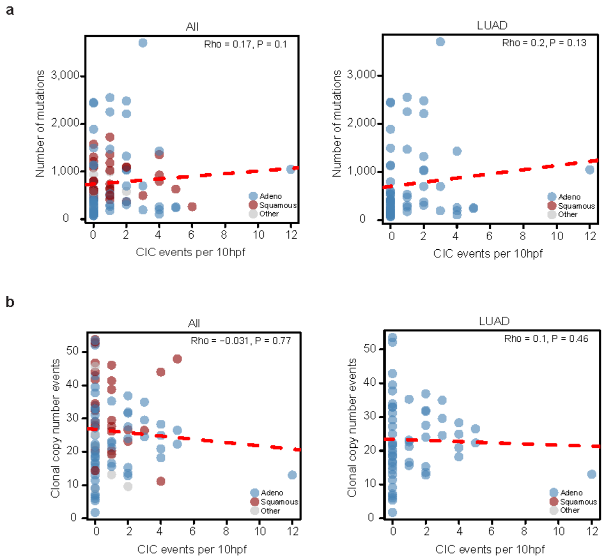

Supplementary Figure 4.
(a) Association between CIC and single nucleotide variation using in ALL (left) and LUAD (right) cases. (b) Association between CIC and clonal copy number events in ALL (left) and LUAD (right). Statistical analysis was Spearman's rank correlation, with rho and p values indicated on the graphs

Supplementary Figure 5

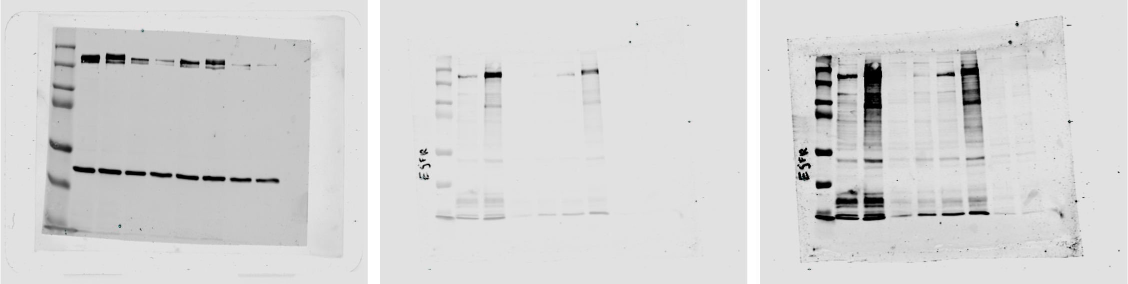

Supplementary Figure 5
Full scan of western blots corresponding to Figure 2j. Left- EGFR (Top, Rabbit – detected in 680nm) and Actin (Bottom, Mouse-detected in 800nm) overlayed in grayscale. Middle- pEGFR (Rabbit- detected in 680 nm) short exposure, Right – pEGFR (Rabbit-detected in 680nm) long exposure. Marker – dual colour precision (Biorad) with marker bands: 250, 150, 100, 75, 50, 37, 25, 20, and 10 kD.

Supplementary Table 1. A summary of the patient cohort under study (n=273)

Variable	Category	Value
		69 (44-86)
**Mean follow-up (range in**		838 (2-2119)
		**Number (%)**
**5-year overall survival rate**		51%
**5-year recurrence-free**		57%
**Sex**	Female	110 (40)
	Male	163 (60)
**Pathological stage**	1	110 (40)
	2	108 (40)
	3	37 (14)
	4	8 (3)
	Missing/ambiguous	10 (4)
**Regional nodal metastasis**	Negative	163 (69)
	Positive	73 (31)
	Missing	37 (14)
**Maximum tumour diameter**	<20mm	68 (25)
	20mm-39mm	117 (43)
	≥40mm	84 (31)
	Missing	4 (2)
**Smoking history**	Never	26 (10)
	Current	77 (28)
	Ex	169 (62)
	Unknown	1 (<1)
**Vascular invasion**	Absent	163 (60)
	Present	75 (27)
	Missing	35 (13)
**Pleural stage**	PL0	154 (60)
	PL1	61 (22)
	PL2/3	33 (12)
	Missing	15 (5)
**Mucinous histology**	No	218 (80)
	Yes	45 (16)
	Missing	10 (4)
**P53 protein overexpression**	Negative	113 (41)
	Positive	96 (35)
	Missing	64 (23)
**CIC status**	Negative	122 (45)
	Positive	151 (55)

Supplementary Table 2. Associations of key predictors of clinicopathological data with recurrence in univariate Cox models and combined in a multivariate model. CIC retains statistical significance after key univariate predictors of tumour recurrence have been incorporated. Nominally significant results are in bold.

	Univariate				Multivariate			
	HR	P	95% CI		HR	P	95% CI	
Tumour size	**1.03**	**0.002**	**1.01**	**1.05**	**1.02**	**0.041**	**1.00**	**1.05**
Nodal status	**2.98**	**0.000**	**1.71**	**5.18**	**2.07**	**0.015**	**1.15**	**3.74**
Vascular Invasion	**2.42**	**0.002**	**1.39**	**4.23**	**1.88**	**0.055**	**0.99**	**3.58**
Pleural stage	**1.54**	**0.021**	**1.07**	**2.23**	1.09	0.717	0.69	1.72
Mucinous histology	**1.93**	**0.036**	**1.05**	**3.58**	**2.07**	**0.033**	**1.06**	**4.02**
CIC (with/without)	**2.15**	**0.010**	**1.20**	**3.85**	**1.98**	**0.025**	**1.09**	**3.60**

Supplementary Table 3 p53 mutations that were detected in 11 of 36 tumours that were sequenced. ACA nr (sample identifier), CDS mutations = coding sequence mutation, AA = Amino Acid mutated

ACA no	CDS mutation	AA	Exon	Type	Trans activity	Residue function	Domain function	Structural motif
17	c.734G>T (Substitution)	R248W	7	missense	Non functional	DNA binding	DNA binding	L2/L3
32	c.463A>C (Substitution)	p.T155P	5	missense	Non functional	Phosphorylation site	DNA binding	NDBL/beta-sheets
82	c.536A>G (Substitution)	p.H179R	5	missense	Non functional	Zn binding	DNA binding	L2/L3
90	c.817C>T (Substitution)	p.R273C	8	missense	Non functional	DNA binding	DNA binding	L1/S/H2
94	c.661G>T (Substitution)	p.E221*	6	nonsense	NA	Exposed	DNA binding	NDBL/beta-sheets
133	c.1001G>T (Substitution)	p.G334V	10	missense	Particllay functional	NA	Tetramerisation	C-term/tetramerisation
2	c.734G>T (Substitution)	R248W	7	missense	Non functional	DNA binding	DNA binding	L2/L3
24	c.796G>T (Substitution)	p.G266*	8	nonsense	NA	Buried	DNA binding	NDBL/beta-sheets
46	c.741_742CC>TT (Complex substitution)	p.G245V	7	missense	Non functional	Buried	DNA binding	L2/L3
61	c.233C>T (Substitution)	p.A78V	4	missense	Functional	NA	SH3-like/Pro-rich	SH3-like/Pro-rich
127	c.706T>C (Substitution)	p.Y236H	7	missense	Non functional	Buried	DNA binding	NDBL/beta-sheets

Supplementary Note 1

Names of TRACERx consortium members and collaborators

Selvaraju Veeriah[1], Justyna Czyzewska-Khan[1], Diana Johnson[1], Joanne Laycock[1], Leticia Bosshard-Carter[1], Rachel Rosenthal[1], Pat Gorman[1], Robert E Hynds[1,3], Gareth Wilson[1,2], Thomas B K Watkins[2], Nicholas McGranahan[1,2], Stuart Horswell[2], Richard Mitter[2], Mickael Escudero[2], Aengus Stewart[2], Peter Van Loo[2], Andrew Rowan[2], Hang Xu[2], Samra Turajlic[2,4], Crispin Hiley[2], Christopher Abbosh[1], Jacki Goldman[2], Richard Kevin Stone[2], Tamara Denner[2], Greg Elgar[2], Sophia Ward[2], Marta Costa[2], Sharmin Begum[2], Ben Phillimore[2], Tim Chambers[2], Emma Nye[2], Sofia Graca[2], Maise Al Bakir[2], Kroopa Joshi[1], Andrew Furness[1], Assma Ben Aissa[1], Yien Ning Sophia Wong[1], Andy Georgiou[1], Sergio Quezada[1], John A Hartley[1], Helen L Lowe[1], Javier Herrero[1], David Lawrence[5], Martin Hayward[5], Nikolaos Panagiotopoulos[5], Davide Patrini[5], Shyam Kolvekar[5], Mary Falzon[5], Elaine Borg[5], Teresa Marafioti[5], Celia Simeon[5], Gemma Hector[5], Amy Smith[5], Marie Aranda[5], Marco Novelli[5], Dahmane Oukrif[5], Sam M Janes[5], Ricky Thakrar[5], Martin Forster[5], Tanya Ahmad[5], Siow Ming Lee[5], Dionysis Papadatos-Pastos[5], Dawn Carnell[5], Ruheena Mendes[5], Jeremy George[5], Neal Navani[5], Asia Ahmed[5], Magali Taylor[5], Penny Shaw[5], Junaid Choudhary[5], Yvonne Summers[6], Raffaele Califano[6], Paul Taylor[6], Rajesh Shah[6], Piotr Krysiak[6], Kendadai Rammohan[6], Eustace Fontaine[6], Richard Booton[6], Matthew Evison[6], Phil Crosbie[6], Stuart Moss[6], Faiza Idries[6], Leena Joseph[6], Paul Bishop[6], Anshuman Chaturved[6], Anne Marie Quinn[6], Helen Doran[6], Angela leek[7], Phil Harrison[7], Katrina Moore[7], Rachael Waddington[7], Juliette Novasio[7], Fiona Blackhall[8], Jane Rogan[7], Elaine Smith[6], Caroline Dive[9], Jonathan Tugwood[9], Ged Brady[9], Dominic G Rothwell[9], Francesca Chemi[9], Jackie Pierce[9], Sakshi Gulati[9], Babu Naidu[10], Gerald Langman[10], Simon Trotter[10], Mary Bellamy[10], Hollie Bancroft[10], Amy Kerr[10], Salma Kadiri, [10] Joanne Webb[10], Gary Middleton[10], Madava Djearaman[10], Dean Fennell[11], Jacqui A Shaw[11], John Le Quesne[11], David Moore[11], Apostolos Nakas[12], Sridhar Rathinam[12], William Monteiro[13], Hilary Marshall[13], Louise Nelson[12], Jonathan Bennett[12], Joan Riley[12], Lindsay Primrose[12], Luke Martinson[12], Girija Anand[14], Sajid Khan[15], Anita Amadi[16], Marianne Nicolson[17], Keith Kerr[17], Shirley Palmer[17], Hardy Remmen[17], Joy Miller[17], Keith Buchan[17], Mahendran Chetty[17], Lesley Gomersall[17], Jason Lester[18], Alison Edwards[18], Fiona Morgan[19], Haydn Adams[19], Helen Davies[19], Malgorzata Kornaszewska[20], Richard Attanoos[21], Sara Lock[22], Azmina Verjee[22], Mairead MacKenzie[23], Maggie Wilcox[23], Harriet Bell[24], Natasha Iles[24], Allan Hackshaw[24], Yenting Ngai[24], Sean Smith[24], Nicole Gower[24], Christian Ottensmeier[25], Serena Chee[25], Benjamin Johnson[25], Aiman Alzetani[25], Emily Shaw[25], Eric Lim[26], Paulo De Sousa[26], Monica Tavares Barbosa[26], Alex Bowman[26], Simon Jordan[26], Alexandra Rice[26], Hilgardt Raubenheimer[26], Chiara Proli[26], Maria Elena Cufari[26], John Carlo Ronquillo[26], Angela Kwayie[26], Harshil Bhayani[26], Morag Hamilton[26], Yusura Bakar[26], Natalie Mensah[26], Lyn Ambrose[26], Anand Devaraj[26], Silviu Buderi[26], Jonathan Finch[26], Leire Azcarate[26], Hema Chavan[26], Sophie Green[26], Hillaria Mashinga[26], Andrew G Nicholson[26, 27], Kelvin Lau[28], Michael Sheaff[28], Peter Schmid[28], John Conibear[28], Veni Ezhil[29], Babikir Ismail[29], Melanie Irvin-sellers[29], Vineet Prakash[29], Peter Russell[30], Teresa Light[30], Tracey Horey[30], Sarah Danson[31], Jonathan Bury[31], John Edwards[31], Jennifer Hill[31], Sue Matthews[31], Yota Kitsanta[31], Kim Suvarna[31], Patricia Fisher[31], Allah Dino Keerio[31], Michael Shackcloth[32], John Gosney[32], Pieter Postmus[32], Sarah Feeney[32], Julius Asante-Siaw[32], Hugo J.W.L. Aerts[33], Stefan Dentro[34], Karl Peggs[1,35,36], Roland F Schwarz[37].

Affiliations

1. Cancer Research UK Lung Cancer Centre of Excellence, University College London Cancer Institute, United Kingdom
2. The Francis Crick Institute, United Kingdom
3. Lungs for Living, UCL Respiratory, University College London, United Kingdom
4. The Royal Marsden Hospital, United Kingdom
5. University College London Hospitals NHS Foundation Trust, United Kingdom
6. University Hospital of South Manchester, United Kingdom
7. Manchester Cancer Research Centre Biobank, United Kingdom
8. Christie NHS Foundation Trust, Manchester, United Kingdom
9. Cancer Research UK Manchester Institute, United Kingdom
10. Heart of England NHS Foundation Trust, Birmingham, United Kingdom
11. Cancer Studies and Molecular Medicine, University of Leicester, United Kingdom
12. Leicester University Hospitals, United Kingdom
13. National Institute for Health Research Leicester Respiratory Biomedical, Research Unit, United Kingdom
14. North Middlesex Hospital, United Kingdom
15. Royal Free Hospital, United Kingdom
16. Barnet Hospital, United Kingdom
17. Aberdeen Royal Infirmary, United Kingdom
18. Velindre Cancer Centre, Cardiff, Wales, United Kingdom
19. Cardiff & Vale University Health Board, Cardiff, Wales, United Kingdom
20. University Hospital Of Wales Heath Park, Cardiff, Wales, United Kingdom
21. Department of Pathology, University Hospital of Wales and Cardiff University, Heath Park, Cardiff, Wales, United Kingdom
22. The Whittington Hospital NHS Trust, United Kingdom
23. Independent Cancer Patients Voice, United Kingdom
24. Cancer Research UK & UCL Cancer Trials Centre, United Kingdom
25. University Hospital Southampton NHS Foundation Trust, United Kingdom
26. Royal Brompton and Harefield NHS Foundation Trust, United Kingdom
27. National Heart and Lung Institute, Imperial College, United Kingdom
28. Barts Health NHS Trust, United Kingdom

29. Ashford and St. Peter’s Hospitals NHS Foundation Trust, United Kingdom
30. The Princess Alexandra Hospital NHS Trust, United Kingdom
31. Sheffield Teaching Hospitals NHS Foundation Trust, United Kingdom
32. Liverpool Heart and Chest Hospital NHS Foundation Trust, United Kingdom
33. Dana-Farber Cancer Institute, Brigham & Women’s Hospital, Harvard Medical School, 450 Brookline Ave, JF518, Boston, MA 02115-5450, USA
34. Wellcome Trust Sanger Institute, Hinxton, CB10 1SA, United Kingdom
35. Cancer Immunology Unit, University College London Cancer Institute, United Kingdom
36. Research Department of Haematology, University College London Cancer Institute, United Kingdom
37. Berlin Institute for Medical Systems Biology, Max Delbrueck Center for Molecular Medicine, Berlin, Germany
