# Supplementary material for: Genomic instability in mutant p53 cancer cells upon entotic engulfment
Source: Nat Commun. 2018 Aug 3;9:3070. doi: 10.1038/s41467-018-05368-1 (PMC6076230; doi:10.1038/s41467-018-05368-1)
Supplement: Supplementary file 2 — Description of Additional Supplementary Files [file 41467_2018_5368_MOESM2_ESM.pdf]

Description of Additional Supplementary Files

File Name: Supplementary Movie 1
Description: Engulfment of a KO 273H/mChery A431 cell (red) by a Ctr 273H/GFP A431 cell (green), resulting in the formation of a CIC structure. As shown, the internalised cell has just undergone mitosis before one daughter is engulfed.

File Name: Supplementary Movie 2
Description: Engulfment of an EV/mCherry cell (red) by a 273H/GFP mutant p53 cell, (green) resulting in the formation of a CIC structure.

File Name: Supplementary Movie 3
Description: Following the CIC formation between the host (red) and internalised cell (green), the internal cell escapes the structure after 8h and the red host cell appears to divide as normal.

File Name: Supplementary Movie 4
Description: Following engulfment, the internalised cell (green) divides within the host cell (red).

File Name: Supplementary Movie 5
Description: Following engulfment, the green internalised cell can be seen to round and bleb after approximately 3 hours, with the red host cell unaffected.

File Name: Supplementary Movie 6
Description: The green host cell in the CIC structure can be sen to bleb and die after 20 hours, the internal green cell also appears to be killed.

File Name: Supplementary Movie 7
Description: The green host cell can be seen appearing to round up for a mitotic division, however the internal green cell disrupts this and only one daughter cell is produced.

File Name: Supplementary Movie 8
Description: The green host cell can be seen to undergo a failed division event. This happens again and subsequently causes the death of the host cell following the unsuccessful tripolar division event.

File Name: Supplementary Movie 9
Description: A failed division event can be seen which fails due to the internalised cell. Subsequently, after release of the internalised cell, a tripolar divion event can be seen with the daughter cells surviving as shown by their ability to divide again.
